# Evidence-Based Nursing Practices for the Prevention of Newborn Procedural Pain in Neonatal Intensive Therapy—An Exploratory Study

**DOI:** 10.3390/ijerph191912075

**Published:** 2022-09-23

**Authors:** Hanna Popowicz, Katarzyna Kwiecień-Jaguś, Wioletta Mędrzycka-Dąbrowska, Monika Kopeć, Danuta Dyk

**Affiliations:** 1 Department of Obstetric and Gynecological Nursing, Medical University of Gdańsk, 80-211 Gdansk, Poland; 2 Department of Anesthesiology Nursing and Intensive Care, Medical University of Gdańsk, 80-211 Gdansk, Poland; 3 Department of Human Nutrition, University of Warmia and Mazury, 10-718 Olsztyn, Poland; 4 Department of Anesthesiology and Intensive Care Nursing, Poznań University of Medical Sciences, 60-806 Poznan, Poland

**Keywords:** evidence-based nursing practice, procedural pain, neonatal intensive therapy

## Abstract

Background: Due to the progress in neonatology, in particular, in the past three decades, the mortality rate among patients of intensive care units has decreased. However, this is connected not only with newborns needing to stay longer in the unit, but also with the exposure of newborns to many painful procedures and stresses. Lack of or insufficient pain prevention has a negative impact on the sensory or locomotor development of newborns. Despite the presence of guidelines based on scientific evidence, the use of pharmacological and non-pharmacological pain-management methods in newborns is still insufficient. Aim: The aim of the study was to: identify the knowledge nurses/midwives have of recommended non-pharmacological and/or pharmacological methods, in particular, in relation to medical intervention procedures; assess the interventions for pain relief applied by midwives/nurses most often in their clinical practice; examine the role of age, general work experience, education level and years of work of medical professionals on a neonatal ward, as well as the referral level of a unit, versus the application of pharmacological and non-pharmacological methods. Methods: A descriptive and quantitative study conducted in 2019 among Polish nurses/midwives working at neonatal intensive care units. Results: The analysis of the material reflected the deficit of knowledge and the insufficient daily use of recommended pain-relief measures among the respondents. Conclusions: The interpretation of data indicates that despite the clear and easily available recommendations of scientific societies concerning the mode of conduct in particular medical procedures, medical personnel do not apply those recommendations in their everyday practice. It is necessary to plan and implement education strategies for nurses/midwives on standard pain-management interventions during painful medical procedures.

## 1. Introduction

The changes implemented in the system of periparturient care, including neonatology, have contributed to the growth of the level of care of patients in intensive care units and have had an efficient impact on the growth of the survival rate among neonates [1,2,3,4]. Unfortunately, this is also often connected with the longer hospitalization of an infant who must undergo many therapeutic, diagnostic and care procedures. The observance in the intensive care unit of principles such as minimal handling, reducing and combining of procedures, the avoidance of noise and strong light, the provision of proper sleep, the reestablishment of ties between parents and their children, as well as pain prevention, not only shortens hospitalization, but also supports the development and improves the quality of life of the child [2,3,4,5].

Undoubtedly, nurses’/midwives’ knowledge of and approach to pain monitoring, treatment and prevention form an important element of the care of a patient in a neonatal intensive care unit [6,7]. At present, medical professionals fully realize that the immature nervous system of a newborn (in particular, a prematurely born neonate) when stimulated by a pain stimulus can contribute to disorders in the development and operation of that system [8,9,10,11,12]. The negligence of pain prevention and relief measures can result in the development of complications in the child’s organism at present and future development stages [6,7,13]. The whole therapeutic team is expected to take action to assess and treat pain complaints and, in particular, take preventive measures during the performance of everyday care and therapeutic procedures. In addition, all medical practice, including the role of nurses, should be based on scientific evidence and existing recommendations. In that way, the patient and the patient’s relatives can be sure that pain evaluation scales and treatment methods will be based on the best and latest guidelines [14].

Nurses/midwives should be aware that patients of intensive care units can experience pain even during the performance of routine nursing activities. They should also use tools to measure pain intensity and skillfully recognize a painful experience and, if necessary, implement pain-relief measures in an adequate way [15,16].

In the opinion of the American Academy of Pediatrics (AAP), the Canadian Pediatric Society (CPS) and the Polish Neonatal Society (PTN), which are leading societies in pediatrics and neonatology, the preparation of pain prevention plans and medical procedures based on the latest scientific research can bring about positive effects in relation to pain monitoring, treatment and prevention in neonatal intensive care units. All of these societies are also of the opinion that it is necessary to implement uniform neonatal care and therapeutic principles and apply well-known and standard pain treatment methods [15,17,18]. In the AAP’s opinion, painkilling actions in intensive care units should include a commonly applicable six-level pain-management plan, which clearly defines the application of individual pain-relief methods depending on the type of pain experience, the painkiller operating mechanism, and relevant procedures [8,15,18]. The foundation of pain prevention in such a specific population of patients hospitalized in neonatal wards is believed to be minimal handling, as well as the application of non-invasive patient-monitoring techniques (e.g., capnography, pulse oximetry) and a reduction in procedures that cause pain. The application of five consecutive levels of the ladder in pain management in neonatal intensive care units allows for effective analgesia in neonates [15,17,18]. The six-level pain-management therapy is described in detail in Table 1.

Knowledge of degrees of pain intensity, pain sources and available pharmacological and non-pharmacological methods in pain management is an important element of pain-prevention procedures [18,19,20]. The literature describes three sources of pain suffered by patients of neonatal intensive care units: procedural, severe and chronic [17,21,22,23]. Scientific studies confirm that patients of neonatal intensive care units usually experience procedural pain caused by care, diagnostic and therapeutic procedures, such as: the administration of vitamin K, vaccination, vein and/or artery puncture, making access to the vena cava, pulmonary toilet, and eye and physical examinations.

It must be taken into account that the intensity of therapeutic and diagnostic intervention felt by this population is dependent on the maturity of the neonate, because children born prematurely are more sensitive and feel pain in a more expressive way because of their immature nervous system [24,25].

### Aim

The aim of the study was to: (1) identify the level of medical professionals’ knowledge of recommended non-pharmacological and/or pharmacological methods in particular medical intervention procedures; (2) evaluate pain-relief interventions applied by midwives/nurses most often in their clinical practice; (3) examine the role of age, general work experience, education level and years of work of medical professionals at a neonatal ward, and the referral level of a unit versus the application of pharmacological and non-pharmacological methods.

## 2. Methods

### 2.1. Design

The research was conducted by use of a descriptive method. The project was carried out in Polish neonatal intensive care units.

### 2.2. Participants

The project was carried out in 2019 and was part of a wider scientific project. The study group included 558 medical professionals (midwives, nurses) employed in neonatal intensive care units classified as secondary and tertiary referral departments (43 Polish hospitals in total).

### 2.3. Data Collection

The project was part of a wider multicenter study focused on the problem of pain-management practices in neonatal intensive care units. A small part of the results was published in 2021 [26]. The initial research and publication focused on nurses’ knowledge and beliefs about neonatal pain. The study was conducted on the basis of a standardized questionnaire tool, which focused on 5 aspects of perceptions of neonatal pain by nurses [26].

In that part of the project, the research team focused on identifying what kind of intervention (pharmacological and non-pharmacological) is used by nurses in their daily practice. Do nurses have practical knowledge about pain management? To do so, the research team used an original questionnaire consisting of three parts.

Part 1: Social and demographic characteristics of nurses and midwives, such as age, education level, general work experience, experience at neonatal intensive care units, the level of referral, and the voivodeship of residence.

Part 2: Identification of the knowledge nurses and midwives have of recommended non-pharmacological and/or pharmacological methods and, in particular, medical procedures. The respondents were requested to answer the following question: “*In your opinion, what actions **are recommended** during the performance of particular procedures*?” They could choose several answers from a range of pharmacological and/or non-pharmacological methods. Having analyzed the available answers, the respondent was to choose one or several answers that were the most relevant in their opinion.

Part 3: Identifying the use of pain-management methods by nurses and midwives to relieve pain in neonates in the case of specific diagnostic and therapeutic interventions. The respondents were requested to answer the following question: “*What actions are usually **taken** at your ward during the performance of particular procedures*?” Based on their practical experience, the respondents were to indicate the three most frequent methods applied to relieve pain in the case of specific medical procedures.

Our survey was developed and analyzed on the basis of the guidelines of international scientific societies (PTN, AAP) [15,17]. The reliability of the questionnaire was verified on the basis of the general Cronbach £—value 0.94. Cronbach’s coefficient level for part 2 was excellent (Cronbach £ 0.90; *p* = 0.005) and for part 3 was good (Cronbach £ 0.85; *p* = 0.005). The questionnaire was supplemented with the information that the respondent takes part in the survey on an anonymous and voluntary basis. The pilot survey was conducted in one of the hospitals in Gdansk among a group of 10 respondents.

### 2.4. Study Procedure

One hundred and twenty hospitals with neonatal intensive care units (secondary and tertiary referral levels) were invited to participate in this project. Out of 52 hospitals, only 43 were included in the study procedure. The selection of hospitals (secondary and tertiary levels) was made on the basis of the European Hospital Reference List [27]. Although all of the medical centers agreed to participate in the project, some respondents and nursing managers refused to participate. This was the main reason why those facilities were excluded from the study. The questionnaire package was delivered to all medical facilities that gave their official consent.

Out of 1039 questionnaires sent to hospitals, 795 questionnaires were returned, including 558 correctly completed forms. Those questionnaires were included in the further statistical analyses.

### 2.5. Inclusion and Exclusion Criteria

- Inclusion criteria: nurses/midwives authorized to perform their profession and working at neonatal intensive care units of secondary and tertiary referral levels; official permission from the CEO/president of a hospital taking part in the study; the respondent’s consent to take part in the project in the form of the voluntary and anonymous completion of the questionnaire.
- The study did not include persons who met any of the following exclusion criteria: a profession other than a nurse/midwife; a nurse/midwife not registered/not working at a neonatal intensive care unit; lack of consent from the hospital management; lack of the respondent’s consent to take part in the project.

### 2.6. Statistical Analysis

Statistical calculations were made by use of the IBM SPSS 23 statistical package and an Excel 2016 spreadsheet. The reliability of the questionnaire was checked with the Cronbach £ level. The qualitative variables are presented in the form of sizes and percentage values, and the quantitative variable in the form of an arithmetic mean and standard deviation. The significance of differences between more than two groups was verified by use of the Kruskal–Wallis test and the one-factor analysis of variance (ANOVA) and between two groups by use of the Student’s *t*-test. In the event of obtaining significant results, the Bonferroni correction test was used. The Kolmogorov–Smirnov test (K-S test) was used to recheck the compliance of the analyzed variables with normal distribution. In all calculations, the significance level of *p* ≤ 0.05 was assumed.

## 3. Results

### 3.1. Sociodemographic Characteristics

**Figure 1:**
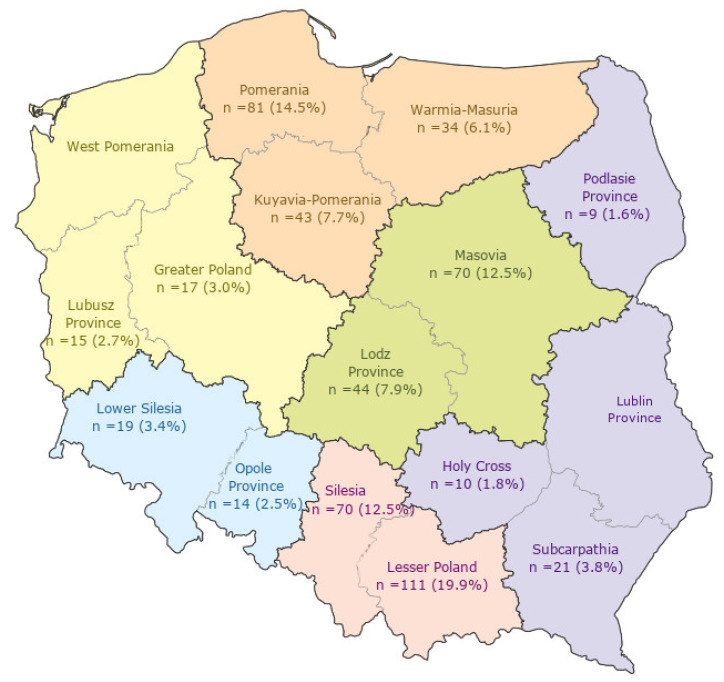
Distribution of the respondents in particular voivodeships.

Out of 120 hospitals invited into the project, only 43 medical centers from various voivodeships in Poland were included in the further analyses. The study covered hospitals of secondary and tertiary referral levels. The greatest number of questionnaires was returned in the Małopolskie Voivodeship (*n* = 111; 19.9%), and the smallest number in the Podlaskie Voivodeship (*n* = 9; 1.6%). Analyzing the age structure of the respondents, 43.7% (*n* = 244) of the nurses and midwives were of 31 to 50 years of age, and 37.5% (*n* = 209) of the respondents had secondary-level medical education (medical college or secondary school). The vast majority of the respondents were employed at hospitals of the secondary referral level (*n* = 340; 60.9%). Over 64.9% (*n* = 362) of the respondents had more than 10 years of professional experience at neonatal intensive care units. The detailed distribution of the respondents is presented in Figure 1 and Table 2.

### 3.2. Medical Professionals’ Knowledge of Recommended Pharmacological and Non-Pharmacological Pain-Management Methods Applied in Medical Procedures

During the study, the respondents were asked about pain-management methods recommended by the PTN in medical procedures such as: heel, finger, vein and artery puncture; cannulation; deep puncture; catheterization of navel vessels; subcutaneous and intramuscular injection; introducing a gastric tube; removing a venous catheter and plaster; lumbar puncture; pulmonary toilet; and intubation (immediately after delivery, mechanical ventilation, and administration of surfactant).

Based on an analysis of the responses, it was confirmed that in the case of heel- and finger-puncture procedures, the vast majority of the respondents knew the recommended non-pharmacological methods of pain relief. An analysis of the detailed responses clearly indicated that in the case of procedures such as: heel puncture (*n* = 471; 84.4%), finger puncture (*n* = 401; 71.9%), artery puncture (*n* = 388; 69.5%), and blood collection via vein puncture (*n* = 470; 84.2%), the respondents believe that the use of mother’s milk or 20% glucose was the most adequate action of those recommended.

In the case of the introduction of a gastric tube, 64.2% of the respondents pointed out that the most recommended method of discomfort mitigation during that procedure was *speaking quietly* (*n* = 364). In the case of the removal of plasters, 61.8% (*n* = 345) of the respondents were of the opinion that a *dummy* is useful.

In the opinion of the respondents, non-pharmacological methods such as *breast milk* or *glucose solution* are strongly recommended in the case of procedures connected with the tearing of tissues, such as subcutaneous and intramuscular injections (s.c.: *n* = 436; 78.1%; i.m.: *n* = 448; 80.3%) and vein puncture, including blood collection (*n* = 470; 84.2%), peripheral puncture (*n* = 455; 81.5%) and central line puncture (*n* = 259; 51.8%). In turn, only a small percentage of the respondents mentioned Emla cream as a recommended pharmaceutical in such medical procedures.

The study reflected that, in the case of the evacuation of fluids from the bronchi system, the responses differed, and almost 42% (*n* = 232) of the respondents believed that *speaking quietly* is the most recommended non-pharmacological method. Moreover, 32% (*n* = 202) of the respondents were of the opinion that *wrapping/FT* should be used. Other responses given by the respondents included: *breast milk or 20% glucose solution* (*n* = 100, 17.9%), *dummy* (*n* = 36, 6.5%), *local anesthesia with lidocaine* (*n* = 10, 1.8%), and *opioids or opioid derivatives* (*n* = 42, 7.5%).

Subsequently, the respondents were requested to indicate which of the actions specified in the questionnaire complied with the PTN’s recommendations and would be taken for pain-prevention purposes during intubation in the case of an emergency or to administer surfactant. An analysis of the detailed responses indicated that medical professionals know that there is no time to apply non-pharmacological pain-relief methods in the case of life emergencies. Every fifth respondent stated that in such a case, as recommended, they would administer *sedatives* (*n* = 116, 20.8%) and *muscle relaxants* (*n* = 107, 19.2%). Medical professionals also chose *speaking quietly* (*n* = 96, 17.2%) as one of the pain prevention methods. In the case of intubation aimed at mechanical ventilation, only 46.6% of the respondents (*n* = 260) chose *sedatives*, while 34.1% chose *muscle relaxants* (*n* = 190) and 32.2% decided that *opioids or opioid derivatives* had to be administered (*n* = 180, 32.3%). Table 3 presents the detailed distribution of responses concerning recommended pain-prevention procedures in neonates.

### 3.3. Application of Recommended Non-Pharmacological and Pharmacological Methods in Clinical Practices by Medical Personnel

During the study, the respondents were asked about pain-management methods recommended by the PTN in medical procedures such as: heel, finger, vein, artery puncture; cannulation; deep puncture; catheterization of navel vessels; subcutaneous and intramuscular injection; introducing a gastric tube; removing a venous catheter and a plaster; lumbar puncture; pulmonary toilet; and intubation (immediately after delivery, mechanical ventilation, and administration of surfactant).

The study reflected that nurses and midwives usually used *mother’s milk* or >20% *glucose* to relieve pain in medical procedures such as heel puncture (*n* = 407; 72.9%), finger puncture (*n* = 330; 59.1%) and the catheterization of navel vessels (*n* = 197; 35.3%).

In the respondents’ opinion, *speaking quietly* is an effective measure that relieves complaints connected with introducing a gastric tube (*n* = 340; 60.9%) and the removal of a cannula from a peripheral vein (*n* = 270; 48.4%), and every second person used non-nutritive sucking while removing plasters.

In turn, *wrapping/facilitated tucking* was the least frequently chosen option by the respondents to relieve discomfort or pain in the above medical procedures, except for nasogastric intubation, where the use of a *dummy* was a procedure that was the least frequently applied by the respondents.

The analysis indicates that the medical professionals most frequently used *breast milk*/>*20% glucose* to mitigate unpleasant feelings connected with lumbar puncture (*n* = 214; 38.4%), blood collection from a vein (*n* = 420; 75.3%), artery puncture (*n* = 313; 56.1%) and cannulation (*n* = 387; 69.4%). In turn, only every fourth respondent stated that they used *Emla cream* in the above cases.

The analysis of the data also indicated that the respondents most frequently used *glucose/breast milk* as a pain-relief method during intramuscular injections (*n* = 348; 62.4%), subcutaneous injections (*n* = 339; 60.8%) and ECC line placement (*n* = 220; 39.4%). A small percentage of the respondents used *Emla cream* as a pharmaceutical product during the above therapies (i.m.—2.9%; s.c.—2.7%; ECC—2.7%). Even fewer respondents stated that they used *lidocaine spray*/gel (i.m.—0.4%; s.c.—0.9%). The results of the study are presented in Table 2.

The analyses confirmed that approximately 33% (*n* = 181) of the respondents believe that to reduce pain while sucking fluids from the bronchi system, *speaking quietly* or *wrapping/FT* are necessary (*n* = 129; 23.1%). In turn, only 4 persons (0.7%) out of 558 stated that they used *lidocaine in ampoules* in that procedure. Pharmaceuticals of the opioid group were administered by no more than 4.8% (*n* = 27) of the respondents.

Regarding the responses given by nurses and midwives to the question concerning the most frequent actions taken during intubation in the case of an emergency, the vast majority answered that they did not use analgesic methods.

However, every seventh respondent answered that in the case of an emergency they administered *sedatives* (13.1%), every eighth also administered *opioids or opioid derivatives* (11.6%), and every tenth gave *muscle relaxants* (10.8%).

In cases where a neonatal patient was intubated for respiratory therapy purposes, medical professionals (*n* = 184; 33%) usually administered *sedatives* and in the case of short-term intubation to give a surfactant, every fifth respondent, on average, usually applied pharmacological methods, including *opioids or opioid derivatives* and *sedatives*, which are not recommended.

In all of the above procedures of neonate intubation, the respondents most frequently used a non-pharmacological method to soothe painful feelings, i.e., *speaking quietly*.

Table 4 presents the responses concerning methods applied to relieve pain in neonates. 

### 3.4. Examination of the Role of Age, General Work Experience, Education Level and Years of Work of Medical Professionals at a Neonatal Ward, and the Referral Level of a Unit versus the Application of Pharmacological and Non-Pharmacological Methods

The data did not have normal distribution. The results were analyzed by the Kruskal–Wallis test, one-factor analysis of variance (ANOVA) and the Student’s *t*-test. In the case of significant results, Bonferroni’s multiple comparison method was applied.

- *Age*

The Kruskal–Wallis test showed that, in the case of the *age* variable, non-pharmacological methods were used mainly by the respondents aged up to 30 and from 31 to 50 (H_(2)_ = 14.7; *p* < 0.05). Between other variables, no statistically significant differences were observed. The detailed results are presented in Table 5.

- *Years of work*

Another variable that was taken into account was the general work experience of medical professionals. The analysis with the Kruskal–Wallis test confirmed that non-pharmacological methods recommended by scientific societies are much more often used by medical professionals having up to 5 and from 6 to 10 years of work experience (H_(2)_ = 6.5; *p* < 0.05) (Table 5).

- *Professional experience*

The impact of professional experience gained at neonatal intensive care units on the use of pain treatment methods was also measured by the use of the Kruskal–Wallis test. The analysis confirmed that statistically significant non-pharmacological methods were used by the respondents who worked at neonatal units for 1 year to 10 years (H_(2)_ = 6.4; *p* < 0.05). Between other variables, no statistically significant differences were observed (Table 5).

- *Education level*

The relation between the use of non-pharmacological/pharmacological methods and the level of education of the respondents was examined by use of one-factor variation analysis (ANOVA). It was confirmed that non-pharmacological methods were much more often used by nurses/midwives with a master’s degree and specialized training (F(2.5) = 5.6, *p* < 0.05). In turn, pharmacological methods were usually used by the respondents with a bachelor’s degree (F(2.5) = 5.2, *p* < 0.05) (Table 6).

- *Level of referral of a unit*

The relation between pain-relief methods and the place of employment and the level of referral was examined by the Student’s *t*-test. The results confirmed that non-pharmacological methods were much more often used by medical professionals at hospitals of a higher referral level (III° (t_(555)_ = 6.1; *p* < 0.001)) in comparison to the respondents working at hospitals of the secondary referral level (Table 7).

## 4. Discussion

The major purpose of the study was to examine whether nurses and midwives know the recommendations of international organizations concerning pain prevention and therapy, as well as which methods are used in practice in neonatal intensive care units in Poland. Contemporary studies indicate that both nurses and physicians know that newborns feel pain and are convinced that there are short-term and long-term unfavorable consequences of untreated pain [26,27].

However, the staff of neonatal intensive care units do not always fully use measures that relieve pain or improve comfort in their medical intervention for various reasons, such as lack of time or an insufficient amount of training [26,28,29]. The guidelines issued by the AAP, CPS and PTN clearly confirm that medical intervention procedures, both pharmacological and non-pharmacological, should improve discomfort and prevent pain experience [15,17]. In addition, the six-level pain prevention ladder to be applied in procedures that generate minor stress or a mild/moderate/strong pain in the specific population of neonates helps to take non-pharmacological and/or pharmacological actions aimed at preventing pain experience [15,17,18]. The analyses confirmed that the respondents had a diversified knowledge of the scope of both (pharmacological/non-pharmacological) recommended medical procedures, and the level of that knowledge was insufficient. Many authors point out that personnel of neonatal hospital wards have no or insufficient knowledge of pain relief in newborns [30,31].

In this study, the pain-relief methods most frequently indicated by the respondents as those used in medical procedures included: *mother’s milk/glucose* and a *dummy*. The outcome of the analysis is similar to the outcome of studies carried out by other authors. Apart from the above methods, Brazilian medical professionals also place a particular emphasis on patient positioning [32].

Undoubtedly, the lack of necessary knowledge on how to use pain-relief procedures in neonates results in a lower quality of neonatal care. Medical professionals should not only know the issues hereunder, but they should also apply them skillfully in their everyday work with patients [33]. The results of the study hereunder indicate that nurses/midwives are neither involved in nor follow proper clinical practices in relation to their patients. Only a small percentage of the respondents stated that they used non-pharmacological methods in all procedures. The analysis of the literature confirmed that the results of the study hereunder are similar to the projects carried out by other, foreign researchers [32,34,35].

The study carried out by Asadi-Noghabi indicated a lack of knowledge of pain-management issues among Iranian nurses. The studies confirmed that the personnel only sometimes used both (pharmacological/non-pharmacological) pain-management methods. In addition, they did not take any pain-relief actions during painful medical procedures such as venous blood collection, intramuscular injections or peripheral punctures [34]. Similarly to the above studies, in another study conducted in Africa, medical professionals also presented poor knowledge of pain prevention in neonates. The deficit of knowledge among nurses/midwives mostly referred to the use of analgesia in various painful medical procedures. In turn, in contrast to Iranian nurses and the respondents of the study hereunder, medical professionals in Africa used non-pharmacological methods in every procedure, for both potentially and actually painful procedures. The most willingly used non-pharmacological procedures included: breast feeding, positioning and non-nutritive sucking. In addition, in comparison with the results of the study hereunder, other authors point out that most respondents use pharmacological agents in medical procedures as one of the main pain-relief methods, e.g., in lumbar puncture, intubation, thorax drainage and central line puncture [35].

In turn, the study performed by Costa et al. indicated that those nurses, unlike the Polish respondents, have good knowledge of non-pharmacological pain-relief measures. Despite this, similarly to the study hereunder, they did not use that knowledge in their clinical practice [32].

The study conducted in Poland and the results thereof confirm that nurses/midwives much more willingly use pain-relief methods in their clinical practice in relation to neonatal patients if they have knowledge of pain issues and pain-management methods in defined painful medical procedures.

The interpretation of the results reveals that the respondents not only have a deficit of knowledge of the recommended pain-relief methods, in particular, for medical procedures, but they are also insufficiently committed to the relevant measures aimed at relieving discomfort and pain in patients during painful medical procedures.

Undoubtedly, the lack or insufficient use of both interventions (pharmacological/non-pharmacological) connected with specific procedures is alarming, in particular, in frequent painful procedures such as heel, vein or artery puncture or pulmonary toilet. It is necessary to implement changes in the organization of pain management in this group of patients, which means involving personnel at all healthcare levels [19,26].

## 5. Study Limitations

Study limitations include the fact that only one professional group was involved—nurses and midwives—as there are also other professional groups which work at neonatal intensive care units and must take pain-prevention measures. Another limitation was the number of hospitals which agreed to participate in the study. The invitation was sent to many hospitals in Poland. Unfortunately, only 52 responded and 43 participated in the project and returned the questionnaires.

## 6. Conclusions

The analyses indicated that it is necessary to plan and implement a strategy of training on standard interventions in pain therapy and prevention during painful medical procedures for nurses/midwives. Indubitably, this would contribute to the multiplication of the frequency of use of both pain-relief methods (pharmacological/non-pharmacological) and improve the quality of pain prevention and treatment in neonates at intensive care units.

## 7. Implications for Practice

This study is one of the first of such projects carried out in Poland. The results point out that it is necessary to plan and implement a strategy of promoting and improving personnel’s awareness of pain-relief methods to be applied in particular medical procedures causing moderate/strong pain in specific populations of patients such as premature infants and neonates. Due to the lack of uniform actions, pain (monitoring, treatment or prevention) is still a great problem at healthcare centers.

Future studies should cover all professional groups to enable a comparison of the organization of pain prevention in various clinical circumstances.

## Figures and Tables

**Table 1:** Six-level pain-management therapy [15,17,18].

Level	Type of Pain	Pharmacological Actions	Non-Pharmacological Actions	Application
**5**	Severe, chronic, long-lasting, moderate-to-strong pain.	Opioids (e.g., morphine, fentanil), sedatives (ketamine) and other tranquilizers, such as midazolam, dexmedetomidine.	Wrapping, massaging, positioning, elements of sensory stimulation.	Inserting a drain into the pleural cavity; surgical interventions; post-surgery pain; wound treatment; taken into consideration in the case of lumbar or central line puncture; intubation and mechanical ventilation.
**4**	Mild-to-moderate procedural pain.	Local infiltration anesthesia (lidocaine, bupivacaine, ropivacaine).	Oral administration of breast milk, glucose/sucrose, kangaroo care, massage, dummy, facilitated tucking, wrapping.	Peripheral and central arterial/venous access device placement; circumcision; inserting a drain into the pleural cavity.
**3**	Chronic/severe, procedural mild-to-moderate pain.	Acetaminophen	Kangaroo care, massage, dummy, facilitated tucking, wrapping.	Post-surgery pain; change of wound dressing; wound treatment; post-circumcision pain.
**2**	Mild/moderate procedural pain.	Local surface anesthetics: lidocaine, Emla cream, lignocaine.	Oral administration of breast milk, glucose/sucrose, kangaroo care, massage, dummy, facilitated tucking, wrapping.	Lumbar puncture; access to veins/arteries; central line placement; intramuscular injection or subcutaneous injection; respiratory physiotherapy; eye examination; pulmonary toilet; intubation; wound treatment.
**1**	Mild, procedural pain.	N/A **	Oral administration of breast milk, glucose/sucrose, kangaroo care, massage, dummy, facilitated tucking, wrapping, sensory stimulation.	Removal of plaster; introducing a gastric tube; catheterization of navel vessels; catheterization of urinary bladder; removing a venous catheter; physical examination; replacement of a nappy. * Heel/finger puncture.
Care foundations		Minimal handling, reduction in the frequency of painful procedures, application of non-invasive patient-monitoring methods (NIRS, pulse oximetry, capnography, patient monitors, transcutaneous bilirubin measurement, etc.).		

Legend: * Please note that heel/finger puncture causes moderate pain and surface anesthetics in this procedure are ineffective, which is why only pharmacological methods are applied. ** N/A—not applicable.

**Table 2:** Sociodemographic characteristics.

Characteristics of the Studied Group		*N*	*%*
**Age**	Less than 30 years	100	17.9
	31–50 years	244	43.7
	More than 50 years	214	38.4
**Level of education**	MSc in nursery/midwifery, specialist in neonatological nursing	178	31.9
	BSc in nursery/midwifery	171	30.6
	Secondary (medical school of nursing or medical college)	209	37.5
**General professional experience**	Up to 5 years	83	14.9
	6–10 years	54	9.7
	More than 10 years	421	75.4
**Professional experience gained at neonatal care units**	Less than 1 year	42	7.5
	1–10 years	154	27.6
	More than 10 years	362	64.9
**The hospital level due to the European Reference Network**	Level II	340	60.9
	Level III	218	39.1

**Table 3:** The distribution of correct answers given by the respondents with respect to pharmacological and non-pharmacological methods applied in the selected medical procedures.

**Finger Puncture**			
	**Correct answers % (*n*)**	**M**	**SD**
**Mother’s milk, >20% glucose**	**71.9 (409)**	**59.3**	**11.0**
**Wrapping/FT**	**45.5 (254)**		
**Speaking quietly**	**57.2 (319)**		
**Non-nutritive sucking**	**62.7 (350)**		
**Heel puncture**			
**Mother’s milk, >20% glucose**	**84.4 (471)**	**68.4**	**12.9**
**Wrapping/FT**	**53.6 (299)**		
**Speaking quietly**	**64.2 (358)**		
**Non-nutritive sucking**	**71.5 (399)**		
**Introducing a gastric tube**			
**Mother’s milk, >20% glucose**	**28.5 (159)**	**41.4**	**20.9**
**Wrapping/FT**	**53.2 (297)**		
**Speaking quietly**	**64.2 (364)**		
**Non-nutritive sucking**	**19.5 (109)**		
**Plaster removal**			
**Mother’s milk, >20% glucose**	**59.1 (330)**	**56.3**	**7.8**
**Wrapping/FT**	**44.4 (248)**		
**Speaking quietly**	**59.7 (333)**		
**Non-nutritive sucking**	**61.8 (345)**		
**Removing a venous catheter**			
**Mother’s milk, >20% glucose**	**57.0 (318)**	**51.2**	**7.8**
**Wrapping/FT**	**39.8 (222)**		
**Speaking quietly**	**54.8 (306)**		
**Non-nutritive sucking**	**54.1 (302)**		
**Catheterization of navel vessels**			
**Mother’s milk, >20% glucose**	**44.3 (247)**	**33.0**	**9.0**
**Wrapping/FT**	**22.4 (125)**		
**Speaking quietly**	**33.0 (184)**		
**Non-nutritive sucking**	**32.4 (181)**		
**Artery puncture**			
**Mother’s milk, >20% glucose**	**69.5 (388)**	**46.0**	**16.6**
**Wrapping/FT**	**35.7 (199)**		
**Speaking quietly**	**45.3 (253)**		
**Non-nutritive sucking**	**53 (296)**		
**Emla cream**	**26.3 (147)**		
**Vein puncture**			
**Mother’s milk, >20% glucose**	**84.2 (470)**	**57.8**	**21.2**
**Wrapping/FT**	**47.8 (267)**		
**Speaking quietly**	**59.9 (334)**		
**Non-nutritive sucking**	**68.8 (384)**		
**Emla cream**	**28.3 (168)**		
**Vein cannulation**			
**Mother’s milk, >20% glucose**	**81.5 (455)**	**53.5**	**20.2**
**Wrapping/FT**	**38.9 (217)**		
**Speaking quietly**	**54.8 (306)**		
**Non-nutritive sucking**	**62.5 (349)**		
**Emla cream**	**29.9 (167)**		
**Lumbar puncture**			
**Mother’s milk, >20% glucose**	**46.4 (259)**	**40.1**	**9.1**
**Wrapping/FT**	**21.9 (122)**		
**Speaking quietly**	**35.3 (197)**		
**Non-nutritive sucking**	**38.4 (214)**		
**Emla cream**	**40.5 (226)**		
**Subcutaneous injection**			
**Mother’s milk, >20% glucose**	**78.1 (436)**	**43.1**	**27.3**
**Wrapping/FT**	**41.9 (234)**		
**Speaking quietly**	**54.3 (303)**		
**Non-nutritive sucking**	**60.9 (340)**		
**Emla cream**	**18.8 (105)**		
**Lidocaine gel/spray**	**4.8 (27)**		
**Intramuscular injection**			
**Mother’s milk, >20% glucose**	**80.3 (448)**	**44.5**	**26.8**
**Wrapping/FT**	**43.2 (241)**		
**Speaking quietly**	**54.3 (303)**		
**Non-nutritive sucking**	**60.2 (336)**		
**Emla cream**	**23.5 (131)**		
**Lidocaine gel/spray**	**5.6 (31)**		
**ECC central puncture**			
**Mother’s milk, >20% glucose**	**51.8 (289)**	**25.4**	**16.2**
**Wrapping/FT**	**23.7 (132)**		
**Speaking quietly**	**33.9 (189)**		
**Non-nutritive sucking**	**35.8 (200)**		
**Emla cream**	**15.8 (88)**		
**Lidocaine gel/spray**	**11.5 (64)**		
**Infiltrated lidocaine**	**5.0 (28)**		
**Bronchial toilet**			
**Mother’s milk, >20% glucose**	17.9 (100)	**18.6**	**16.7**
**Wrapping/FT**	36.2 (202)		
**Speaking quietly**	**41.6 (232)**		
**Non-nutritive sucking**	**6.5 (36)**		
**Lidocaine**	**1.8 (10)**		
**Opioids and opioid derivative**	**7.5 (42)**		
**Intubation for mechanical ventilation purposes**			
**Opioids and opioid derivative**	**32.3 (180)**	**29.7**	**17.5**
**Muscle relaxants**	**34.1 (190)**		
**Sedatives**	**46.6 (260)**		
**Atropine**	**5.6 (31)**		
**Endotracheal intubation to administer surfactant**			
**Opioids and opioid derivative**	**27.2 (152)**	**17.5**	**11.6**
**Muscle relaxants**	**20.6 (115)**		
**Atropine**	**4.3 (24)**		

Legend: M—percentage mean value of all interventions correctly chosen by the respondents in a given procedure; SD—standard deviation.

**Table 4:** The distribution of answers given by the respondents with respect to pharmacological and non-pharmacological methods applied in the selected medical procedures.

**Finger Puncture**			
	**Correct Answers % (*n*)**	**M**	**SD**
**Mother’s milk, >20% glucose**	**59.1 (330)**	49.3	11.6
**Wrapping/FT**	**34.1 (190)**		
**Speaking quietly**	**46.4 (259)**		
**Non-nutritive sucking**	**57.5 (321)**		
**Heel puncture**			
**Mother’s milk, >20% glucose**	**72.9 (407)**	58.8	12.9
**Wrapping/FT**	**43.9 (245)**		
**Speaking quietly**	**53 (296)**		
**Non-nutritive sucking**	**65.2 (364)**		
**Introducing a gastric tube**			
**Mother’s milk, >20% glucose**	20.8 (116)	35.3	20.4
**Wrapping/FT**	42.5 (237)		
**Speaking quietly**	**60.9 (340)**		
**Non-nutritive sucking**	17 (95)		
**Plaster removal**			
**Mother’s milk, >20% glucose**	41.8 (233)	40.7	11.6
**Wrapping/FT**	24.4 (136)		
**Speaking quietly**	**51.4 (287)**		
**Non-nutritive sucking**	45.3 (253)		
**Removing a venous catheter**			
**Mother’s milk, >20% glucose**	40.7 (227)	37.5	12.4
**Wrapping/FT**	19.7 (110)		
**Speaking quietly**	**48.4 (270)**		
**Non-nutritive sucking**	41.4 (231)		
**Catheterization of navel vessels**			
**Mother’s milk, >20% glucose**	**35.3 (197)**	21.5	9.8
**Wrapping/FT**	**11.8 (66)**		
**Speaking quietly**	**19.5 (109)**		
**Non-nutritive sucking**	**19.5 (109)**		
**Artery puncture**			
**Mother’s milk, >20% glucose**	**56.1 (313)**	31.6	20
**Wrapping/FT**	22.9 (128)		
**Speaking quietly**	37.5 (209)		
**Non-nutritive sucking**	39.1 (218)		
**Emla cream**	2.5 (14)		
**Vein puncture**			
**Mother’s milk, >20% glucose**	**75.3 (420)**	45	26.7
**Wrapping/FT**	34.6 (193)		
**Speaking quietly**	52 (290)		
**Non-nutritive sucking**	58.4 (326)		
**Emla cream**	**5 (28)**		
**Vein cannulation**			
**Mother’s milk, >20% glucose**	**69.4 (387)**	41.2	5.7
**Wrapping/FT**	33.2 (185)		
**Speaking quietly**	43 (240)		
**Non-nutritive sucking**	54.5 (304)		
**Emla cream**	**5.7 (32)**		
**Lumbar puncture**			
**Mother’s milk, >20% glucose**	**38.4 (214)**	25	9.4
**Wrapping/FT**	12 (67)		
**Speaking quietly**	24.4 (136)		
**Non-nutritive sucking**	23.5 (131)		
**Emla cream**	26.5 (148)		
**Subcutaneous injection**			
**Mother’s milk, >20% glucose**	**60.8 (339)**	32.2	25.5
**Wrapping/FT**	30.5 (170)		
**Speaking quietly**	48 (268)		
**Non-nutritive sucking**	50 (279)		
**Emla cream**	2.7 (15)		
**Lidocaine gel/spray**	**0.9 (5)**		
**Intramuscular injection**			
**Mother’s milk, >20% glucose**	**62.4 (348)**	32.7	27.7
**Wrapping/FT**	30.1 (168)		
**Speaking quietly**	50 (279)		
**Non-nutritive sucking**	50.5 (282)		
**Emla cream**	2.9 (16)		
**Lidocaine gel/spray**	0.4 (2)		
**ECC central puncture**			
**Mother’s milk, >20% glucose**	**39.4 (220)**	14.9	13.7
**Wrapping/FT**	16.1 (90)		
**Speaking quietly**	19.5 (109)		
**Non-nutritive sucking**	21.5 (120)		
**Emla cream**	2.7 (15)		
**Lidocaine gel/spray**	3.6 (20)		
**Infiltrated lidocaine**	1.3 (7)		
**Bronchial toilet**			
**Mother’s milk, >20% glucose**	12.2 (68)	12.7	12.6
**Wrapping/FT**	23.1 (129)		
**Speaking quietly**	**32.4 (181)**		
**Non-nutritive sucking**	3 (17)		
**Infiltrated lidocaine**	0.7 (4)		
Opioids and opioid derivative	4.8 (27)		
**Intubation for mechanical ventilation purposes**			
**Opioids and opioid derivative**	24.2 (135)	20.5	12.5
**Muscle relaxants**	21.5 (120)		
**Sedatives**	33 (184)		
**Atropine**	3.2 (18)		
**Endotracheal intubation to administer surfactant**			
**Opioids and opioid derivative**	24.2 (126)	13.8	9.8
**Muscle relaxants**	15.6 (87)		
**Atropine**	3.2 (18)		

Legend: M—percentage mean value of all interventions correctly chosen by the respondents in a given procedure; SD—standard deviation.

**Table 5:** Age, general professional experience, and professional experience gained at NICU vs. non-pharmacological and pharmacological actions.

Non-Pharmacological Actions vs. Age	*N*	M	SD	H	Df	*p*
Less than 30 years	100	16.8	6.0			
31–50 years	244	16.2	6.4	14.7	2	0.001 *
More than 50 years	214	14.8	6.2			
**Pharmacological** **actions vs. age**						
Less than 30 years	100	4.3	3.7			
31–50 years	244	3.5	3.3	4.5	2	0.107
More than 50 years	214	4.1	4.0			
**Non-pharmacological actions vs. general experience**						
Up to 5 years	83	16.7	5.9			
6–10 years	54	17.1	6.5	6.5	2	0.038 *
More than 10 years	421	15.1	6.5			
**Pharmacological** **Actions vs. general experience**						
Up to 5 years	83	4.4	3.7			
6–10 years	54	3.8	3.4	3.3	2	0.189
More than 10 years	421	3.8	3.7			
**Non-pharmacological actions vs. professional experience at the NICU**						
Less than 1 year	42	15.0	6.8			
1–10 years	154	16.8	6.0	6.4	2	0.041 *
More than 10 years	361	15.0	6.5			
**Pharmacological actions vs. professional experience at the NICU**						
Less than 1 year	42	3.8	3.7			
1–10 years	154	4.2	4.0	1.6	2	0.443 *
More than 10 years	361	3.8	3.5			

Legend: Kruskal–Wallis test (H statistic); M—mean; SD—standard deviation; df—degrees of freedom; * statistically significant *p* < 0.05.

**Table 6:** Educational level vs. non-pharmacological and pharmacological actions.

Non-Pharmacological Actions	*N*	M	SD	F	df	*P*
MSc in nursery/midwifery, specialist in neonatological nursing	178	16.8	6.0			
BSc in nursery/midwifery	171	15.0	6.2	5.6	2.5	0.004 *
Secondary (medical school of nursing or medical college)	209	14.7	6.8			
**Pharmacological Actions**	***N***	**M**	**SD**	**F**	**df**	***P***
MSc in nursery/midwifery, specialist in neonatological nursing	178	3.6	3.1			
BSc in nursery/midwifery	171	4.6	4.6	5.2	2.5	0.006 *
Secondary (medical school of nursing or medical college)	209	3.5	3.0			

Legend: ANOVA test (F statistic); M—mean; SD—standard deviation; df—degrees of freedom; * statistically significant *p* < 0.05.

**Table 7:** Non-pharmacological and pharmacological actions vs. referral level.

Non-Pharmacological Actions	*N*	M	SD	t	df	*P*
Level II	330	14.2	6.0			
Level III	227	17.5	6.6	6.1	555	0.000 *
**Pharmacological Actions**	***N***	**M**	**SD**	**t**	**df**	***P***
Level II	330	3.9	4.1			
Level III	227	3.9	2.8	0	555	0.974

Legend: Student’s *t*-test (H statistic); M—mean; SD—standard deviation; df—degrees of freedom; * statistically significant *p* < 0.05.

## Data Availability

The data that support the findings of this study are available from the corresponding author (H.P.) upon reasonable request.
